# Audit of Antibiotic Prescribing Practices for Neonatal Sepsis and Measurement of Outcome in New Born Unit at Kenyatta National Hospital

**DOI:** 10.1155/2019/7930238

**Published:** 2019-04-28

**Authors:** Priti Jagdishbhai Tank, Anjumanara Omar, Rachel Musoke

**Affiliations:** Department of Paediatrics and Child Health, School of Medicine, University of Nairobi, Kenya

## Abstract

**Background:**

Neonatal sepsis is a leading cause of morbidity and mortality globally. A high index of suspicion is required since features of sepsis are nonspecific. Auditing of antibiotic use is necessary to reduce misuse and minimise development of antibiotic resistance.

**Objectives:**

To assess the antibiotic prescribing practices in NBU at KNH against recommended Kenyan guidelines for neonatal sepsis. In addition, outcome within 7 days was described.

**Methods:**

This was a prospective audit of 320 neonates over a 2-month period at NBU of KNH. Data were collected using a structured questionnaire, stored in MS-EXCEL, and analysed using STATA.

**Results:**

Documentation of perinatal risk factors and clinical features at admission and at the time of change of antibiotics was very poor. The rate of investigations to confirm infection was very low. Blood cultures were done only in 13 (4%) neonates on admission, while complete blood count and C reactive protein were done in 224 (70%) and 198 (62%), respectively. Appropriate antibiotics as per the Kenyan guidelines were prescribed in 313 (97.8%) of neonates on admission. However, these were not stopped at 48-72 hours for the 148 (53.62%) who had improved. Overall mortality was high in neonates at 80 (25%). Majority (55%) died within 48 hours. Mortality was high among preterm neonates; 70 (43.8%) died out of 160.

**Conclusion:**

Overall documentation and investigations to confirm infection was poor. The continuation of antibiotics was inappropriate. Overall mortality was high especially in the first 48 hours of admission. To improve documentation, availability of a checklist on admission is recommended.

## 1. Introduction

According to Global Health Observatory data, 2.6 million neonates died in 2016. The main causes of neonatal deaths are prematurity and low birth weight, infections, asphyxia, and birth trauma. Sepsis accounts for around one-third deaths in neonates worldwide [1]. Survivors of neonatal sepsis are at higher risk of neurodevelopmental impairment [2–5].

Neonatal sepsis usually has nonspecific presentation, hence, delay in treatment, and especially initiation of effective antibiotic therapy results in serious consequences ranging from neurodevelopmental deficits to death [6]. Therefore, clinicians are urged to start empiric antibiotics to symptomatic neonates or neonates at high risk of sepsis while awaiting culture results [7]. Judicious use of antibiotics can be life-saving; however, both broad-spectrum antibiotics and prolonged treatment with empiric antibiotics can lead to development of antimicrobial resistance [8, 9]. Prolonged duration of antibiotics can be associated with adverse outcomes like necrotising enterocolitis, late-onset sepsis, and death [10]. Antibiotic prescribing practices should be evaluated periodically for its rational use to prevent emergence of resistance [8].

In an effort to reduce neonatal mortality rate, Ministry of Health of Kenya published Basic Paediatric Protocols (February-2016), which has guidelines for management of neonatal sepsis [11]. Training of health personnel on use of the Kenyan guidelines is done through the emergency triage and treatment plus (ETAT+) course. Adherence to guidelines in terms of choice and duration of antibiotics is very necessary in order to reduce development and spread of resistance in the hospital, as well as in the community [8, 12]. Antibiotics are the most frequently prescribed medicines in newborn unit (NBU) at Kenyatta National Hospital (KNH) but audit of use has not been done. The primary objective of the current study was to assess the antibiotic prescribing practices in NBU at KNH against recommended Kenyan guidelines for neonatal sepsis.

## 2. Methodology

This was a prospective audit carried out over a period of 2 months in the Newborn Unit of Kenyatta National Hospital. KNH is a national tertiary referral and teaching hospital and the NBU has some facility for critical care. As a general rule all neonates with birth weight below 2000g are admitted while those above that weight are only admitted if they require special care. About 30% of all admissions are referrals from other health facilities. The average admission rate is about 250 neonates per month.

**Figure 1:**
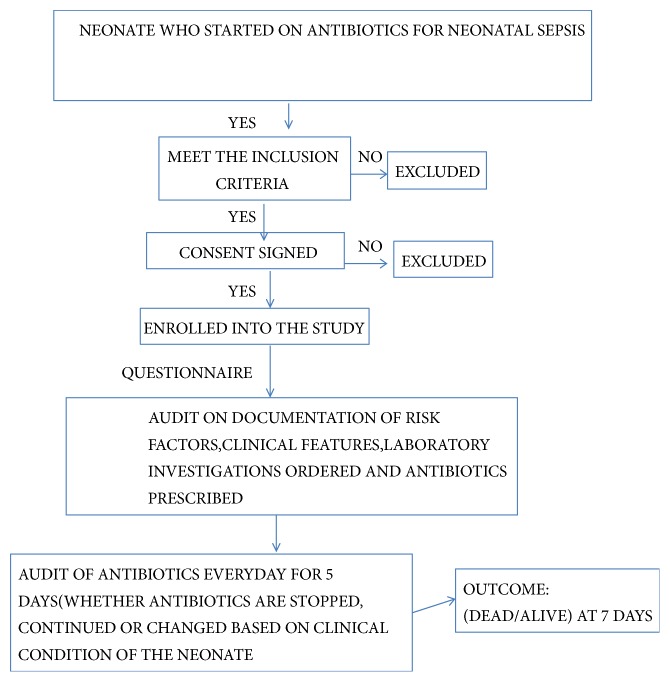
Study flowchart.

The sample size was determined using Fischer's Formula and a sample size of 320 was taken. Population correction factor was applied for finite population. Data were collected daily from the neonate's medical record by PJT with assistance of two clinical officers using a standardised questionnaire. Consecutive sampling of neonates who were started on any antibiotics within the NBU was done. Neonates with birth asphyxia, major congenital malformations, and referred neonates who had received antibiotics for >48 hours were excluded. Collected data were recorded in the computer storage program MS-EXCEL at the end of 7th day of follow-up of the enrolled participant. Data verification was done manually for each record by PJT. To prevent inappropriate use access to the data required a password.  Figure 1 shows the flow of data collection.

### 2.1. Statistics

Data analysis was done by STATA software package. Continuous variables were summarised using means (Standard Deviation) and medians (range). Categorical variables were presented as frequency distributions using tables or graphs. We computed the proportion of neonates whose prescription conforms to the national recommendations. The data collected on the audit of antibiotic prescribing practices were compared with the recommended Kenyan guidelines. The chi square test was used to compare the outcome variable (dead or alive at 7 days) versus categorical variables across two groups by level of guideline adherence. All independent variables were combined to generate regression models to compare effect on the outcome variable. The proportion of correct antibiotic prescribing practices determined 95% confidence interval. The proportion of neonates dead or alive at 7 days or on discharge determined 95 % confidence interval.

### 2.2. Ethics

A written informed consent was obtained from the parent/guardian. Approval was obtained from the KNH/UON ethics committee.

## 3. Results

### 3.1. Sociodemographic Characteristic of Study Population

A total of 320 neonates aged 0 day to 28 days admitted were enrolled in the study. The median age at admission was 1 day (IQR 1-7 days) and 297 (92.8%) were aged between 1 and 3 days. Neonates who received antibiotics for presumed early-onset sepsis were 297 (92.8%), while 23 (7.2%) for late-onset sepsis.  Table 1 shows sociodemographic characteristics of 320 enrolled neonates. It was noted that there was equal distribution between preterm and term neonates.

### 3.2. Audit of Documentation of Clinical Features

Complete documentation of clinical features for neonatal sepsis was not done in any of the 320 neonates. The clinical features not documented by clinician on admission were convulsions in 252 (41.05%), grunting in 128 (20.85%), lower chest wall indrawing in 76 (12.4%), and lethargy in 65 (10.6%) as shown in Table 2. Signs of respiratory distress were mostly present and documented, because study population included preterm neonates as well. Refusal to breastfeed was documented in a few term neonates admitted from postnatal wards of KNH.

### 3.3. Audit of Documentation of Perinatal Risk Factors

Overall documentation of perinatal risk factors was very poor. As mentioned in Table 3, the most commonly documented perinatal risk factors were low birth weight (documented in 100%), prolonged rupture of membranes (documented in 51.6%), suspected or confirmed chorioamnionitis (documented in 21.8%) and difficult or prolonged labour (documented in 7.8%). Maternal fever was documented only in 1.6%. Only 2(0.6%) had history of maternal intrapartum antibiotics use. Overall 53(16.6%) neonates had maternal risk factors present.

### 3.4. Audit of Investigations Done on Admission

Blood cultures were done only in 13(4%) neonates on admission, while complete blood count and C reactive protein were done in 224(70%) and 198(62%) respectively as shown in Table 4. Blood sugar was measured in 26(8%) neonates. Immature to total neutrophil count and lumbar puncture were not done in any of the neonates.

### 3.5. Audit of Antibiotics Prescribing Practices

According to Kenyan guidelines (Basic Pediatrics Protocols – Feb 2016) recommended first-line treatment for neonatal sepsis is penicillin and gentamicin in combination. Appropriate antibiotics as per the Kenyan guidelines were prescribed in 313 (97.8%) of neonates on admission. Ceftazidime and amikacin were prescribed in 7 neonates, while ceftazidime was prescribed for 1 neonate.

Appropriate doses of penicillin and gentamicin were given in 310 (96.9%) and 282 (88%), respectively, on admission. The dose of gentamicin for neonates weighing < 2kgs and aged <7 days is 3mg/kg. Overdose of gentamicin was observed in such babies.

According to Kenyan guidelines (Basic Paediatrics Protocols - Feb 2016), empiric antibiotics should be given for 48-72 hours and may be stopped if the neonate remained entirely well during this period.  Table 5 shows antibiotic usage over 5 days.

*At 48 hours*, out of 276, 148 (53.62%) neonates improved clinically, yet antibiotics were stopped only in 8 (2.9%) and were changed to oral antibiotics in 6 (2.17%). Thirty-four 34 (12.32%) neonates deteriorated clinically and antibiotics were changed for 16 (5.8%). Blood culture and CRP was done for 12 (3.4%).

*At 72 hours, *out of 258, 168 (65.12%) neonates improved clinically, yet antibiotics were stopped only in 22 (8.53%) and were changed to oral antibiotics in 8 (3.11%). Clinical deterioration was noted in 32 (12.4%) neonates and antibiotics were changed for 13 (5.03%). Blood culture was done for 10 (3.9%), while CRP was done for 16 (6.2%).

The number of neonates decreases from 320 to 300 at 24 hours, to 276 at 48 hours, to 258 at 72 hours, and to 214 at 96 hours, and decreasing to 156 at 120 hours, either because the neonates died or discharged. The neonates were followed till discharge or death if it happened before 7 days.

### 3.6. Audit of Outcome at 7 Days

**Figure 2:**
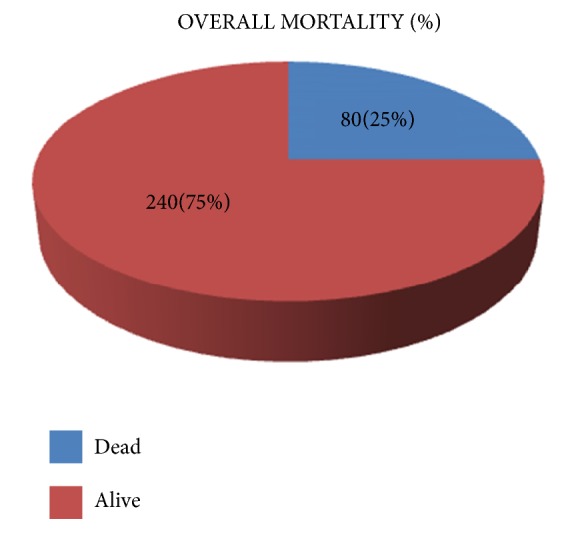
Overall mortality rate.

Overall mortality among 320 neonates admitted was 80 (25%) over 7 days as shown in Figure 2. Mortality was high among preterm neonates; 70 (43.8%) died out of 160.

**Figure 3:**
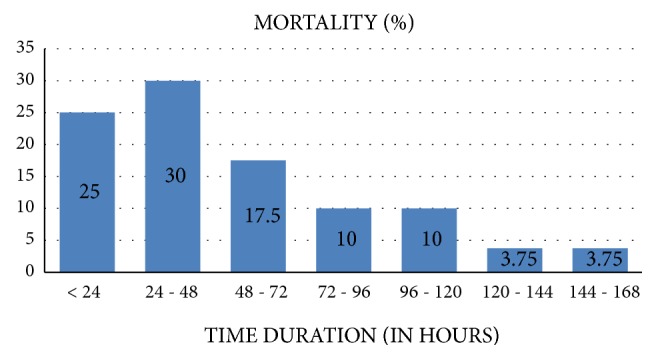
Mortality rate according to time duration.

Figure 3 shows mortality rate according to time duration. Majority 44 (55%) died within the first 48 hours and 36 (45%) died between 48 hours and 7 days. Out of the 44 neonates who died within 48 hours, 38 (86.4%) were preterm.

## 4. Discussion

This study was carried out to audit antibiotic prescribing practices for neonatal sepsis against recommended Kenyan guidelines in the New Born Unit of Kenyatta National Hospital. This was the first audit study done in this unit after the Kenyan guidelines were revised in Feb – 2016.

Complete documentation of clinical features and perinatal risk factors for neonatal sepsis was not done in any of the 320 neonates. These features are clearly listed in the Basic Paediatric Protocols and should have been documented if there was continuous availability of a structured neonatal admission record. A structured neonatal admission form is in existence at KNH but availability had been erratic. It was presumed that some clinical features like convulsions, lethargy, and refusal to breastfeed were only documented if they were present. This appears to be a common Kenyan problem as reported by Kithinji et al. in the KNH general paediatric wards and [13] and Aluvaala et al, in 22 public hospitals of Kenya [14].

Diagnosis of neonatal infection largely depends on laboratory work-up. A full blood count and immature to total neutrophil ratio (I/T ratio) and C-reactive protein are some of the most available tests in KNH. Unfortunately, I/T ratio is rarely done even when requested. Reagents for CRP are also sometimes out of stock. Our 4% rate of blood cultures was much lower than 14% reported by Simiyu et al. in an earlier study from the same NBU [15]. We did not audit availability of blood culture bottles during the current study but it could have contributed to the low levels reported. This is a recurrent problem in our institution. It is also possible that the attending clinician just did not consider doing this procedure. Ideally a neonate who is not responding to antibiotics should be reevaluated but the rate in our study was very low. The same reasons given above could be operational here. In Simiyu et al. study change of antibiotics was guided by culture and sensitivity reports in only 13.5% [15].

As for the blood sugar estimation the NBU is equipped with a glucometer and most of the times the strips for the test are available. This test therefore should have been done more frequently than reported in the current study if the admitting clinician ordered it.

In our study, appropriate antibiotics as per the Kenyan guidelines (Basic Pediatrics Protocols- 2016) were prescribed in 97.8% of neonates on admission. Appropriate doses of penicillin and gentamicin were given in 96.9% and 88%, respectively, on admission. Overdose of gentamicin was observed in neonates weighing <2kgs and aged <7 days. Our findings were better than Kithinji's study in KNH paediatric wards, where the recommended first line antibiotics were given in 64.4% of neonates, with 37.5% of them having error in the dosages. She also reported gentamicin overdosing in neonates <7 days and <2kgs [13]. Aluvaala et al. also found error in dosages; about one in 10 of benzyl penicillin prescriptions was an overdose (11.6%) in contrast to almost one in five (18.5%) for gentamicin [14].

From this study we found that there was prolonged unnecessary use of antibiotics in neonates who improved clinically at 48 – 72 hours just like in other studies done in high resource settings. Neonates who improved clinically at 48 hours were 53.62%, yet antibiotics were stopped in 2.9% only. At 72 hours 65.12% neonates improved clinically, but antibiotics were stopped only in 8.53%. The continuation of antibiotics was more inappropriately done than initiation of antibiotics. Earlier discontinuation of antibiotics was an issue, maybe because of inability to confirm infection. Other studies have reported similar results to ours.

Slogrove et al. in South Africa reported that 33.3% of their infants were asymptomatic and treated empirically; the continuation of treatment beyond 24 hours was probably unnecessary in 38% of early and 28% of late antibiotic events without abnormal inflammatory markers at 48-72 hours. Neonates treated for > 6 days during their first antibiotic event were more likely to require a second antibiotic event (p < 0.02) [16]. Schellack et al. in South Africa also showed overuse of antibiotics; 58% of the antibiotics were administered for 10 days or longer [17]. An audit done by Abdelrhim et al., in United Kingdom in 2012, demonstrated all 22 could have been managed safely with 36hrs of antibiotics, but they were given antibiotics for >48hrs[18]. A Prospective Surveillance done by Cantey et al. in 2011-12 in Texas concluded that 94% of antibiotic use was empiric therapy for suspected infection. Only 63% were discontinued after 48 hours, when cultures were sterile and 26% of antibiotics were continued for ≥5 days despite sterile cultures; pneumonia (16%) and culture-negative sepsis (8%) were the major contributors [19]. A study done by Afjeh SA et al. in Iran also showed prolonged duration of antibiotic for 7-62 days with a mean duration of 24.01 days [20].

A retrospective cohort analysis of extremely low birth weight infants done by Cotton et al. concluded that the median duration of initial empirical antibiotic treatment varied significantly among centres, from 3 to 9.5 days (P < .001). In 27%-85% of neonatal centres empiric antibiotics had been administered for >5 days to neonates with negative cultures [21]. A multicentre study by Cordero and Ayers also reported that most neonates with suspected sepsis and negative blood cultures were given antibiotics, but no perinatal risk factors or clinical signs explained prolonged administration. Discontinuing empiric antibiotics when blood cultures are negative in asymptomatic extremely low birth weight infants can reduce antimicrobial exposure without compromising clinical outcome [22].

We should emphasize on discontinuing empiric antibiotics as soon as is feasible; if the neonate is clinically stable, blood cultures are negative and CRP is normal. There is a lack of well-designed clinical trials evaluating the appropriate duration of empirical antibiotics in blood-culture-negative sepsis. The only randomised controlled trial done by Saini et al. showed that there was no difference in the treatment failure (defined as reappearance of signs of sepsis within 15 days of stopping antibiotics, supported by laboratory evidence) rates between short course (48-96 hours) and long course (7 days) groups among neonates >30 weeks and >1000 grams with probable sepsis [23]. Randomised controlled trial by Gathwala et al. concluded that 10-day antibiotic therapy was as effective as 14-day therapy in blood culture-proven neonatal sepsis, if the neonate is clinically stable by day 7 of treatment [24].

In this study overall mortality was 80 (25%) over 7 days. Of these 70 (87.5%) were preterm neonates. Forty-four (55%) neonates died within 48 hours. It is probable that most of these deaths were due to unavailability of respiratory support. Even though the NBU has facility for continuous positive airway pressure (CPAP) and mechanical ventilation, the numbers requiring this support outstrips availability. This is similar to what was reported by Musoke et al. in year 1992 even though at that time respiratory support was not available [25]. Simiyu et al. in 2004 in NBU at KNH reported a higher mortality rate of 57.4%[15]. Were et al. in KNH- NBU demonstrated neonatal survival rate of 62.6% in neonates born below 2000 grams. None of the neonates born less than 1000 grams survived the neonatal period. They reported that over 28% of the mortality occurring within the first 24 hours, which is comparable to our study, could be due to lack of intensive care facilities and inadequate obstetric services [26].

A prospective study done in Egypt showed mortality rate of 51% for proven early-onset sepsis and 42.9% for proven late-onset sepsis [27]. A prospective study by Baqui et al. in India also reported high mortality in prematurity. In their study, 74.8% of deaths occurred due to prematurity in the first week of life, with 30% in the first 24 hours, and >50% of neonatal deaths secondary to sepsis occurred in the first week of life [28].

## 5. Study Limitation

This study being an audit of medical records only reflected what was documented. Also unavailability of the structured neonatal admission record for all admissions as clinicians only reported the positive responses. Hawthorne effect: people might have changed their practices when they came to know about the study.

## 6. Conclusion

There was poor documentation of clinical features, perinatal risk factors, and condition of the neonates at the time of change of antibiotics. Appropriate antibiotics as per the Kenyan guidelines were given in 97.8% of neonates on admission. The rate of investigations to confirm infection was very low. Blood cultures were done only in 4% of neonates on admission and lumbar punctures were not done. The continuation of antibiotics was inappropriate. Overall mortality was high in neonates at 80 (25%). Most neonates died within 48 hours and majority were preterm.

## Figures and Tables

**Table 1:** Socio-demographic characteristics of participants.

Variable	Characteristics	Frequency (%) n=320
Gestational age(in weeks)	<28	17 (5.3)
	28 – <32	31 (9.7)
	32 - <37	112 (35)
	37- 40	157 (49.1)
	>42	3 (0.9)

Age at admission(in days)	1 – 3	297(92.8)
	≥4	23(7.2)

Sex	Male	170 (53.2)
	Female	150 (46.8)

Birth weight(in grams)	< 1000	16 (5)
	1000 - <1500	36 (11.3)
	1500 - <2500	110 (34.4)
	2500 - <4000	150 (46.8)
	≥4000	8 (2.5)

Referral from another facility	Yes	134 (42)
	No	186 (58)

Place of delivery	Hospital	307 (96)
	Home	13 (4)

Mode of delivery	SVD	147 (46)
	C/S	160 (50)
	Breech	13 (4)

**Table 2:** Audit of documentation of clinical features.

Clinical features(not documented)	Frequency % (n=320)
Convulsions	252(41.1)
Lethargy	65(10.6)
Bulging fontanel	35(5.7)
Lower chest wall indrawing	76(12.4)
Grunting	128(20.9)
Cyanosis	24(3.9)
Pallor	18(2.9)
Jaundice	16(2.6)

**Table 3:** Audit of documentation perinatal risk factors.

Perinatal risk factors present at birth Frequency (n=320) (%)	Documented		Not documented
	Present	Absent	
Maternal fever >38°c	3(1)	2(0.6)	315(98.4)
Suspected or confirmed Chorioamnionitis	30(9.4)	40(12.4)	250(78.2)
Prolonged rupture of membranes	11(3.4)	154(48.2)	155(48.4)
Difficult or prolonged labour	9(2.8)	16(5)	295(92.2)
Received intrapartum antibiotics	2(0.6)	0	318(99.4)
Low birth weight <2500gms	155(48.4)	165(51.6)	0

**Table 4:** Audit of investigations done on admission.

Investigations	Frequency (%) n =320
Complete blood count	224(70)
C reactive protein	198(62)
Immature to total neutrophil count	0
Blood culture	13(4)
Lumbar puncture	0
Blood sugar	26(8)

**Table 5:** Audit of antibiotic usage over 5 days.

	Frequency (%)	At 24 hrs	At 48 hrs	At 72 hrs	At 96 hrs	At 120 hrs
		(n=300)	(n=276)	(n=258)	(n=214)	(n=156)
Condition of the baby clinically	Improved	103(34.3)	148(53.62)	168(65.12)	140(65.4)	114(73.1)
	Deteriorated	29(9.7)	34(12.32)	32(12.4)	24(11.21)	12(7.7)
	No change	148(49.3)	83(30.06)	40(15.5)	35(16.36)	20(12.8)
	Not documented	20(6.7)	11(4)	18(6.98)	15(7.01)	10(6.4)

Antibiotic usage	Stopped	1(0.35)	8(2.9)	22(8.53)	34(15.9)	40(25.65)
	Continued	298(99.3)	246(89.13)	215(83.33)	156(72.9)	106(67.95)
	Changed	0	16(5.8)	13(5.03)	16(7.5)	5(3.2)
	Changed to oral	1(0.35)	6(2.17)	8(3.11)	8(3.7)	5(3.2)

CRP done	Yes	0	12(3.4)	16(6.2)	10(4.7)	6(3.8)
	No	300(100)	264(96.6)	242(93.8)	204(95.3)	150(96.2)

Blood culture done	Yes	0	12(3.4)	10(3.9)	8(3.7)	6(3.8)
	No	300(100)	264(96.6)	248(96.1)	206(96.3)	150(96.2)

## Data Availability

The data used to support the findings of this study are available from the corresponding author upon request.

## Additional Points

*Recommendations.* This study sets a baseline for continued audit and change of practice in the NBU. To improve documentation, a structured neonatal admission record should be available at all times. Adequate follow-up of all babies on antibiotic as per the Kenyan guidelines is needed.
